# Intestinal Obstruction Secondary to Cecal Volvulus as a Result of Intestinal Nonrotation: A Case Report

**DOI:** 10.7759/cureus.89205

**Published:** 2025-08-01

**Authors:** Mohamed Ahmed, Ahmed Allawi, Jami Zajicek, Sarmad Mohammed Salih, Kim Nguyen

**Affiliations:** 1 Surgery, AdventHealth Tampa, Tampa, USA; 2 Surgery, University of California, Riverside, USA; 3 General Surgery, Östersund General Hospital, Ostersund, SWE; 4 Surgery, Oxford University Hospitals NHS Foundation Trust, Oxford, GBR; 5 Colorectal Surgery, AdventHealth Tampa, Tampa, USA

**Keywords:** adult intestinal malrotation, colon obstruction, intestinal nonrotation, ladd’s bands, small intestinal obstruction

## Abstract

One of the extremely uncommon manifestations of malrotation is nonrotation, a condition that arises due to the complete failure of the midgut to rotate 270 degrees. The true prevalence in adults remains obscure due to the scarcity of the condition. A standard surgical approach to management is therefore lacking, and an increasing number of case reports is required to establish evidence-based recommendations. We present the case of a 39-year-old male with acute abdominal pain due to cecal volvulus in the setting of intestinal nonrotation, successfully managed with a robotic-assisted surgical approach.

## Introduction

Intestinal nonrotation is a rare variant of malrotation, a congenital anomaly arising from the failure of the midgut to complete its normal 270-degree counterclockwise rotation during embryologic development [1]. This condition results in an abnormal configuration of the bowel within the abdominal cavity, as described by Fischer et al. [2]. Although it is estimated to affect approximately 2% of the pediatric population [3], the true prevalence in adults remains largely undefined due to its frequently asymptomatic nature and incidental discovery. Clinical manifestations in adults can vary widely - from chronic nonspecific symptoms to acute surgical emergencies - posing significant diagnostic and management challenges. This diagnostic ambiguity, combined with the lack of standardized guidelines, underscores the need for further research to optimize clinical outcomes. In this paper, we report the case of a 39-year-old male who presented with acute abdominal pain secondary to cecal volvulus in the context of intestinal nonrotation. The patient underwent successful robotic-assisted surgical intervention, illustrating the feasibility and advantages of advanced minimally invasive techniques in managing this complex anatomic and surgical scenario [4].

## Case presentation

A 39-year-old, active, Middle Eastern male with no significant past medical history and no prior abdominal surgeries presented to the emergency department with a one-day history of acute-onset, severe, diffuse colicky abdominal pain. The pain was worsened by movement and accompanied by nausea, though the patient denied any episodes of vomiting. He reported his last bowel movement was the day prior to presentation. The patient endorsed a history of occasional abdominal fullness and increased flatulence but denied any prior episodes of similar severe abdominal pain. He also had no history of colonoscopy, inflammatory bowel disease, or other known gastrointestinal conditions. On physical examination, his blood pressure (BP) was 115/64, pulse rate (PR) of 77, respiratory rate (RR) of 20, temperature of 37°C, and oxygen saturation of 98% on room air. He exhibited generalized abdominal tenderness with signs concerning for peritonitis, including voluntary guarding and rebound tenderness. Initial laboratory evaluation revealed a serum lactate of 4 mmol/L, raising concern for bowel ischemia or early sepsis (see Table 1).

**Table 1:** Laboratory values of the patient on the day of presentation to the emergency unit. Abbreviations: BUN, blood urea nitrogen; EGFR, estimated glomerular filtration rate; A/G Ratio, albumin-to-globulin ratio; AST, aspartate aminotransferase; ALT, alanine aminotransferase; WBC, white blood cell (count); RBC, red blood cell (count); MCV, mean corpuscular volume; MCH, mean corpuscular hemoglobin; MCHC, mean corpuscular hemoglobin concentration; RDW, red cell distribution width; MPV, mean platelet volume

Parameter	Value	Units
Glucose, POC	109.00	mg/dL
Sodium	136.00	mmol/L
Potassium	4.10	mmol/L
Chloride	100.00	mmol/L
Carbon Dioxide	21.00	mmol/L
Anion Gap	15.00	mmol/L
BUN	7.00	mg/dL
Creatinine	0.80	mg/dL
BUN/Creatinine Ratio	8.80	ratio
EGFR	115.50	mL/min/1.73 m²
GLUCOSE	92.00	mg/dL
Calcium	9.50	mg/dL
Albumin	3.90	g/dL
Globulin	2.90	g/dL
A/G Ratio	1.30	ratio
Protein, Total	6.80	g/dL
AST	18.00	U/L
ALT	13.00	U/L
Bilirubin, Total	0.90	mg/dL
Alkaline Phosphatase	57.00	U/L
WBC	10.61	10³/uL
RBC	4.58	10⁶/uL
Hemoglobin	14.00	g/dL
Hematocrit	41.60	%
MCV	90.80	fL
MCH	30.60	pg
MCHC	33.70	g/dL
RDW	12.60	%
Platelet Count	210.00	10³/uL
MPV	10.70	fL
Neutrophils %	79.30	%
Lymphocytes %	10.40	%
Monocytes %	8.40	%
Eosinophils %	0.80	%
Basophils %	0.50	%
Immature Granulocytes	0.60	%
Neutrophils Absolute	8.42	10³/uL
Lymphocytes Absolute	1.10	10³/uL
Monocytes Absolute	0.89	10³/uL
Eosinophils Absolute	0.09	10³/uL
Basophil Absolute	0.05	10³/uL
Lactate	3.38	mmol/L

**Figure 1:**
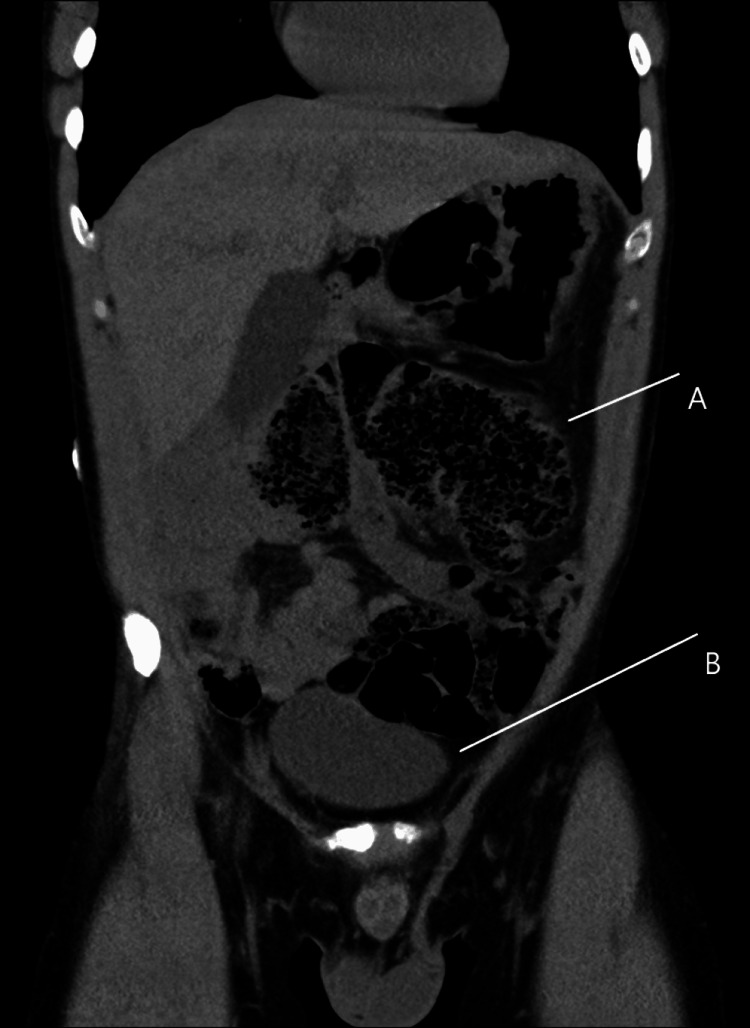
Abdominal CT, cross section, lower abdomen. Coronal section of an abdominal CT showing (A) abnormally positioned distended cecum in the mid- and left abdominal cavity and (B) displaced urinary bladder secondary to bowel distension.

Computed tomography (CT) of the abdomen and pelvis demonstrated features consistent with intestinal nonrotation, characterized by an abnormal positioning of the small bowel predominantly on the right side and the colon on the left. In addition, there were classic radiologic signs of cecal volvulus, including a markedly dilated cecum displaced to the left upper quadrant, a whirl sign indicating mesenteric twisting, and a “bird's beak” tapering at the site of torsion, as illustrated in Figure 1.

The patient was promptly resuscitated with intravenous fluids, resulting in initial symptom stabilization. A contrast-enhanced CT scan of the abdomen and pelvis revealed a mispositioned cecum located in the left mid-abdomen, along with swirling of the adjacent mesenteric vessels, raising suspicion for a partial cecal volvulus. Notably, there was no radiographic evidence of bowel obstruction or ischemia at the time of imaging. Given the high clinical concern for evolving bowel ischemia in the context of malrotation anatomy, a decision was made to proceed with emergent robotic-assisted exploratory surgery (Video 1).

**Video 1:** Nonrotation of the bowel, robotic resection of the transverse colon. A 39-year-old male presented with abdominal pain. Imaging showed bowel obstruction and a malpositioned cecum in the abdominal midline and to the left.

Following successful detorsion of the volvulized bowel, a right hemicolectomy with primary intracorporeal ileocolic anastomosis was performed using the robotic-assisted approach. The procedure was completed without intraoperative complications. The patient’s postoperative course was uneventful. He tolerated advancement of diet appropriately, demonstrated return of bowel function, and reported progressive improvement in symptoms. He was discharged home on postoperative day three in stable condition. At his one-month outpatient follow-up, the patient remained asymptomatic, with no evidence of recurrent abdominal pain, infection, or bowel dysfunction. He continued to recover well and had resumed normal daily activities.

## Discussion

Intestinal rotational disorders affect approximately 0.2%-1% of the general population, though only one in 2,500 individuals develops clinically significant symptoms [5]. The embryologic development of the gastrointestinal tract is a highly regulated process occurring in three distinct phases: physiological herniation of the midgut into the umbilical cord, return of the midgut into the abdominal cavity with a 270-degree counterclockwise rotation around the superior mesenteric artery (SMA), and fixation of the midgut to the posterior abdominal wall. Disruption in any of these stages can result in rotational abnormalities, which most commonly present during infancy and early childhood. Among these, defects during the second phase (rotation of the midgut) lead to conditions such as intestinal malrotation, nonrotation, or reversed rotation [6]. In malrotation, the midgut incompletely rotates, a well-documented condition in the pediatric population. In contrast, intestinal nonrotation occurs when the midgut re-enters the abdominal cavity without undergoing any rotational movement, resulting in an abnormal anatomic configuration - with the small intestine located entirely on the right side and the colon predominantly on the left. A failure of peritoneal fusion between the visceral and parietal peritoneum on the right can lead to cecal hypermobility, creating a loose mesenteric attachment. This anatomical predisposition allows the cecum to migrate and twist, leading to cecal volvulus [7]. Clinical manifestations of nonrotation range from asymptomatic incidental findings to chronic intermittent abdominal pain or even acute bowel obstruction. Timely recognition is crucial to prevent life-threatening complications such as midgut volvulus [8]. Radiologic imaging, especially computed tomography (CT), is instrumental in diagnosing malrotation and nonrotation [9]. While upper gastrointestinal contrast studies remain the gold standard in pediatric cases, CT is more commonly used in adults. Hallmark CT findings include abnormal duodenojejunal junction positioning, the SMV lying to the left of the SMA - a reversal of the normal anatomic relationship [10,11]. The standard of care for symptomatic malrotation at any age is surgical intervention, most commonly via Ladd’s procedure [12]. Although asymptomatic nonrotation may not necessitate immediate treatment, surgical correction is warranted in symptomatic cases or when complications such as volvulus, with or without obstruction, occur [13]. Our case represents, to our knowledge, the first reported successful management of cecal volvulus in the setting of intestinal nonrotation using a robotic-assisted surgical approach. This contribution adds meaningful insight to the sparse existing literature and highlights the utility of minimally invasive techniques in treating complex congenital gastrointestinal anomalies in adults.

## Conclusions

Intestinal nonrotation is a rare congenital anomaly that is often diagnosed incidentally or during evaluation of patients presenting with recurrent abdominal pain and bilious vomiting. In symptomatic cases, particularly those complicated by left-sided cecal volvulus or atypically located appendicitis, computed tomography (CT) plays a critical role in timely and accurate diagnosis. A solid understanding of gut embryology, including the phases of malrotation and nonrotation, is essential for any surgeon managing such complex presentations. While open, laparoscopic, and robotic surgical approaches are all viable options, the robotic-assisted technique has emerged as a safe and effective method, offering enhanced visualization and precision in anatomically distorted fields. Given the rarity of this condition and variability in presentation, continued research and larger case series are necessary to develop evidence-based, standardized surgical management guidelines for intestinal nonrotation in adults.
